# Characterization of More Selective Central Nervous System Nrf2-Activating Novel Vinyl Sulfoximine Compounds Compared to Dimethyl Fumarate

**DOI:** 10.1007/s13311-020-00855-0

**Published:** 2020-05-11

**Authors:** Karl E. Carlström, Praveen K. Chinthakindi, Belén Espinosa, Faiez Al Nimer, Elias S. J. Arnér, Per I. Arvidsson, Fredrik Piehl, Katarina Johansson

**Affiliations:** 1 Department of Clinical Neurosciences, Section of Neurology, Karolinska Institutet, 17177 Stockholm, Sweden; 2 Catalysis and Peptide Research Unit, University of KwaZulu Natal, Durban, 4000 South Africa; 3 Department of Medicinal Chemistry, Drug Design and Discovery, Uppsala University, Box 574, 75123 Uppsala, Sweden; 4 Department of Medical Biochemistry and Biophysics, Division of Biochemistry, Karolinska Institutet, 17177 Stockholm, Sweden; 5 Department of Medical Biochemistry and Biophysics, Science for Life Laboratory, Drug Discovery and Development Platform and Division of Translational Medicine and Chemical Biology, Karolinska Institutet, 171 21 Solna, Sweden; 6 Pfizer Innovation AB, 19190 Sollentuna, Sweden

**Keywords:** Nrf2, NFκB, HIF, dimethyl fumarate, multiple sclerosis, redox regulation, pTRAF, sulfoximine, traumatic brain injury, microglia

## Abstract

The Nrf2 transcription factor is a key regulator of redox reactions and considered the main target for the multiple sclerosis (MS) drug dimethyl fumarate (DMF). However, exploration of additional Nrf2-activating compounds is motivated, since DMF displays significant off-target effects and has a relatively poor penetrance to the central nervous system (CNS). We de novo synthesized eight vinyl sulfone and sulfoximine compounds (CH-1–CH-8) and evaluated their capacity to activate the transcription factors Nrf2, NFκB, and HIF1 in comparison with DMF using the pTRAF platform. The novel sulfoximine CH-3 was the most promising candidate and selected for further comparison *in vivo* and later an experimental model for traumatic brain injury (TBI). CH-3 and DMF displayed comparable capacity to activate Nrf2 and downstream transcripts *in vitro*, but with less off-target effects on HIF1 from CH-3. This was verified in cultured microglia and oligodendrocytes (OLs) and subsequently *in vivo* in rats. Following TBI, DMF lowered the number of leukocytes in blood and also decreased axonal degeneration. CH-3 preserved or increased the number of pre-myelinating OL. While both CH-3 and DMF activated Nrf2, CH-3 showed less off-target effects and displayed more selective OL associated effects. Further studies with Nrf2-acting compounds are promising candidates to explore potential myelin protective or regenerative effects in demyelinating disorders.

**Electronic supplementary material:**

The online version of this article (10.1007/s13311-020-00855-0) contains supplementary material, which is available to authorized users.

## Introduction

The ability to manage oxidative and xenobiotic stress is a highly conserved process engaging several redox-regulated transcription factors [1], both during disease and in the healthy state [2]. Among these, nuclear factor (erythroid-derived 2)-like 2 (Nrf2) is a well-characterized responder to oxidative stress via Kelch-like ECH-associated protein 1 (Keap1), and has been ascribed therapeutic relevance in various cell types during different pathologies [3–6]. In addition, Nrf2 has been suggested a potential therapeutic target during inflammatory conditions [7–9].

One striking example of successful clinical development of Nrf2-modulating drugs is use of dimethyl fumarate (DMF/Tecfidera™) which currently is a leading first-line treatment for relapsing remitting multiple sclerosis (RRMS) worldwide [10–12]. However, as DMF also engages additional transcription factors, it is unclear to what degree therapeutic effects can be associated with activation of solely Nrf2 [13–16]. As an example, apart from affecting Nrf2-regulated transcripts, DMF in RRMS patients also induces transcripts regulated by Nuclear factor κ-light-chain-enhancer of activated B cells (NFκB) and hypoxia-inducible factor (HIF) [17]. In addition, data on blood–brain barrier (BBB) penetrance by DMF, especially in human, is limited which may affect its therapeutic potential and motivates further research on other types of Nrf2 activators [18]. In line with this, there is a growing interest for novel Nrf2-activating compounds and especially vinyl sulfone and sulfoximine compounds in the field of drug discovery [19–21].

The aim of this study was to synthesize and characterize more selective Nrf2-activating compounds able to target CNS-resident cells. Expression patterns were evaluated *in vitro* and *in vivo* following stimulation with DMF or one of eight synthesized compounds (CH-1–CH-8). The vinyl sulfoximine CH-3 was selected for further *in vivo* studies in naïve rats and in an experimental model for traumatic brain injury (TBI), which is known to activate of Nrf2, NFκB, and HIF1 pathways, as well as displaying cell infiltration [16], focal immune cell activation [22], and neurodegeneration [4, 20].

## Methods

### Transcriptional Response Analysis

Assessing the transcriptional activities of Nrf2, NFκB, and HIF1 was done using the pTRAF tool as described previously [14]. Generation of stable HEK(pTRAF^Nrf2/HIF/NFkB^) reporter cell line were described previously (Sthijns MMJPE et al, 2017). Stably transfected HEK(pTRAF^Nrf2/HIF/NFkB^) reporter cells were seeded in a collagen I-coated 96-well plates at a density of 18 000 cells/well and incubated overnight, followed by exposure of different transcription factor inducers as indicated (Thermo Fisher Scientific, Waltham, MA) [41]. Samples were loaded in as technical duplicates or triplicates. For microscopy, cell nuclei were stained with 40 ng/ml Hoechst for 30 min and subsequently fixated in 2% ice-cold paraformaldehyde for 10 min at RT. The Operetta High Content Imaging System with the Columbus System was used to analyze images and determine the mean fluorescence intensity per cell.

### *In Vivo* Experiments

Dark Agouti (DA) rats were bred in the in-house breeding facility of the Karolinska University Hospital and fed standard rodent chow and water ad libitum. Animals were kept in open cages in a facility with 12-h light/dark cycles. Animals were given vehicle CH-3 (25 mg/kg) or DMF (25 mg/kg) in 0.1% methyl cellulose via oral gavage in a total volume of 1 mL (Sigma, St. Louis, MO). For temporal assessments after single doses, groups of three animals were used for every time point. TBI was performed on 10–12-week-old DA males under deep isoflurane anesthesia using a weight-drop model [23]. Rats were placed in a stereotactic frame and injected with 250 μL bupivacaine 2.5 mg/mL (Sigma, St. Louis, MO) under the skin. The skull bone was exposed and a small hole was drilled 3 mm posterior and 2.3 mm lateral of bregma. A focal cerebral contusion injury was made using a weight-drop device hitting a piston resting on the dura matter [24]. The piston was allowed to vertically dislocate the dura matter 3 mm in the vertical axis. Any local hemorrhage was stopped and the skin was closed using sutures. Animals were given CH-3 (25 mg/kg) (*n* = 7) or DMF (25 mg/kg) (*n* = 7) or only vehicle (*n* = 6) as described in Fig. 4(D). All experiments were approved and performed in accordance with Swedish National Board of Laboratory Animals and the European Community Council Directive (86/609/EEC) under the permits N275-15 and N244-13.

### Flow Cytometric Analysis

Rats were perfused with 100 mL PBS, and the brain was removed and minced using a sterile scalpel. The tissue was incubated in 1 mL Accutase and 4 μL DNase/mL at + 37 °C (Sigma, St. Louis, MO) and passed through a fire-polished Pasteur pipette every 10th minute, this was repeated three times with decreasing pipette opening. The homogenate was filtered through a 70-μm strainer and myelin was removed by a 37% Percoll (Sigma, St. Louis, MO) layer spun at 800×*g* for 10 min at + 10 °C without additional acceleration or brake. The pellet was re-suspended to a single cell suspension and stained for anti-O4, anti-Cd11b, and anti-Cd45 (Novus Biologicals, Littleton, CO). Dead cells were excluded using near IR Live/Dead probes (Thermo Fisher Scientific, Waltham, MA). Samples were analyzed with a 3-laser Beckman Coulter Gallios using Kaluza Software.

### Neurofilament Light Protein

Rat cerebrospinal fluid (CSF), about 100 μL, was collected directly after sacrifice, prior to PBS perfusion, from the cerebellomedullary cistern and directly frozen and stored at − 70 °C. Concentrations of neurofilament light protein (NF) were determined using the NF-Light ELISA exactly according to the manufacturer’s instructions (Umandiagnostics, Umeå, Sweden). Samples were diluted 1:3 and loaded in duplicates.

### Cell Cultures

HEK293 cells and stable HEK(pTRAF^Nrf2/HIF/NFkB^) reporter cells were cultured in Eagle’s minimum essential medium, supplemented with 10% fetal bovine serum and 100 U penicillin/ml and 100 μg streptomycin/ml. Cells were cultured at 37 °C in a humidified atmosphere with 5% CO_2_ and 21% O_2_. Primary rat cultures were established from neonatal pups. Microglia and oligodendrocytes were isolated as described for flow cytometry; following Percoll layering, cells were labeled with either anti-cd11b or anti-A2B5 Microbeads (Milteny Biotec, Bergisch, D). Microglia were cultured in DMEM/F12 supplemented with 100 U penicillin/ml and 100 μg streptomycin/ml and 5% fetal bovine serum (Thermo Fisher Scientific, Waltham, MA). Oligodendrocytes were cultured in in 200 mL Neurobrew, 100 mL N2-supplement (Milteny Biotec, Bergisch, D), 2 mL bFGF (PeproTech, Princeton, NJ), and 2 mL PDGF-BB (R&D Systems, Minneapolis, MA) per 10 mL of DMEM/F-12 media on poly-l-lysine pre-coated culture ware. For differentiation of oligodendrocytes, bFGF and PDGF-BB was removed 48 h prior to stimulation.

### Quantitative Real-Time PCR

Total RNA was isolated from rat tissue or human or rat cells using RNeasy mini kit (Qiagen, Venlo, Netherlands) exactly as described by the manufacturer’s instructions, including 15 min on-column DNase digestion. cDNA was prepared with reverse transcriptase PCR using iScript kit (BioRad Laboratories, Hercules, CA). Amplifications were conducted using Bio-Rad SYBR green according to the manufacturer’s instructions and plates were run in Bio-Rad CFX optical system, and samples were loaded in triplicates (BioRad Laboratories, Hercules, CA). Primers were designed to be optimal at + 60 °C and to span an exon-exon junction using online software at http://www.ncbi.nlm.nih.gov.

#### Human primers:

- *HPRT*: F: CTCATGGACTGATTATGGACA; R: GCAGGTCAGCAAAGAACTTAT
- *B-ACTIN*: F: CATGTACGTTGCTATCCAGGC; R: CTCCTTAATGTCACGCACGAT
- *GSTA4*: F: TCAGCTGAGCCTTGCAGATGTGA; R: GGGGGAGGCTTCTTCTTGCTGC
- *GCLM*: F: CATTTACAGCCTTACTGGGAGG; R: ATGCAGTCAAATCTGGTGGCA
- *TXN*: F: ATATGGCAAGAAGGTGATGCTCC; R: CGTGGCTGAGAAGTCAACTACTA
- *VEGF*: F: TCTGCAGCTCTGTGTGAAGG; R: ACTTCTCCACAACCCTCTGC
- *TXNDC17*: F: GCAGGCTGAACCAGTCGTA; R: TACCAGTTTTTGAGGTGTTCCATA
- *iNOS*: F: ACAAATTCAGGTACGCTGTG; R: TGCACGAGCCTGTAGTG
- *IL8*: F: TCTGCAGCTCTGTGTGAAGG; R: ACTTCTCCACAACCCTCTGC
- *NQO1*: F: AGTGCAGTGGTGTGATCTCG; R: GGTGGATCACGCCTGTAAT
- *TXNRD1*: F: ATATGGCAAGAAGGTGATGCTCC; R: GGGCTTGTCCTAACAAAGCTG
- *HMOX1*: F: CCGACAGCATGCCCCAGGATT; R: GTCTCGGGTCACCTGGCCCTT

#### Rat Primers

- *Hprt*: F: CTCATGGACTGATTATGGACA; R: GCAGGTCAGCAAAGAACTTAT
- *B-actin*: F: CGTGAAAAGATGACCCAGATCA; R: AGAGGCATACAGGGACAACACA
- *Gclm*: F: AGTGGGCACAGGTAAAACCC; R: ACTTGCCTCAGAGAGCAGTTC
- *Nqo1*: F: CAGAAACGACATCACAGGGGA; R: GGCCTTCCTTATACGCCAGA
- *Vegf*: F: GGGAGCAGAAAGCCCATGAA; R: GCTGGCTTTGGTGAGGTTTG
- *iNos*: F: CAACATCAGGTCGGCCATTACT; R: TAGCCAGCGTACCGGATGA
- *Il6*: F: AGAAAAGAGTTGTGCAATGG; R: ACAAACTCCAGGTAGAAACG
- *Txn*: F: GTAGACGTGGATGACTGCCA; R: CTCCCCAACCTTTTGACCCTT
- *Gsta4*: F: CAGGAGTCATGGAAGTCAAAC; R: TTCTCATATTGTTCTCTCGTCTC
- *Ark1b8*: F: GACTTCCAGTTGAGCGACCA; R: TCCATGTTGACTGTCTCAGGC
- *Hmox1*: F: AGGGAAGGCTTTAAGCTGGT; R: AGGGAAGTAGAGTGGGGCAT
- *Bax*: F: TTGCTACAGGGTTTCATCCAGG; R: CACTCGCTCAGCTTCTTGGT

### Statistical Analysis

General statistical analyses were performed in GraphPad Prism software. Principal component analyses and hierarchy clustering were generated in R using ggbiplot. Comparisons between two groups were done with Student’s two-tailed unpaired *t* test. Comparisons between paired groups were done with paired *t* test. Two group comparisons with a control group were done with one-way ANOVA with Benjamin-Hochberg post-hoc test. *P* < 0.05 was throughout considered statistically significant.

## Results

### Synthetization of the VSC2(CH-1) Analogues CH-2–CH-8

**Fig. 1:**
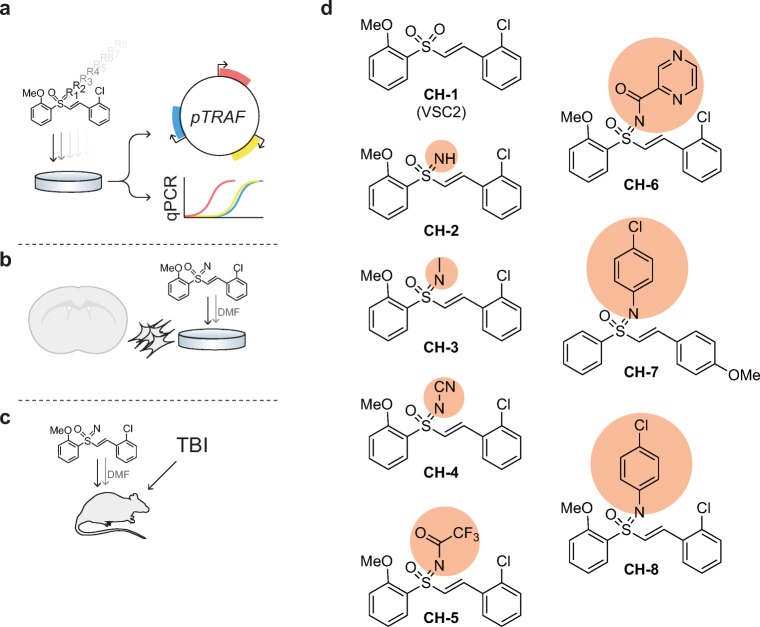
Experimental outline and structures of newly synthetized compounds. (A) Initial screening of compound CH-1–8 and DMF using pTRAF and qPCR. (B) Stimulation of primary glial cultures with DMF and CH-3. (C) Evaluation of CH-3 and DMF in an experimental model for traumatic brain injury. (D) Chemical structure of CH-1–8

Vinyl sulfone compounds, have been implicated as modulators of Nrf2 activity. Herein we first evaluated the vinyl sulfone compound VSC2 ((E)-1-(2-((2-methoxyphenyl)sulfonyl)vinyl)-2-chlorobenzeneanalogues)/CH-1, which has been suggested to activate Nrf2-regulated pathways [19–21] (Fig. 1(A, D), Supplementary Table 1). Exchange of the –O atom (i.e. sulfone CH-1) with a –NH moiety yields a novel sulfoximine (CH-2) (Fig. 1(D)); such isosteric replacement may improve compound quality [25] as well as activity [26]. We thus synthetized sulfoximine compounds depicted in Fig. 1(D). Based on our earlier synthetic methodology reports, we have synthesized CH-2 [27] and carried out *N*-functionalization such as *N*-methylation (CH-3) [28], *N*-cyanation (CH-4) [29], *N*-arylation (CH-7, 8) [30], and *N*-acylation (CH-5, 6) (Fig. 1(D), Supplementary Table 1).

### CH-3 Is More Specific to Nrf2 Compared to DMF in pTRAF-Transfected HEK293 Cells

**Fig. 2:**
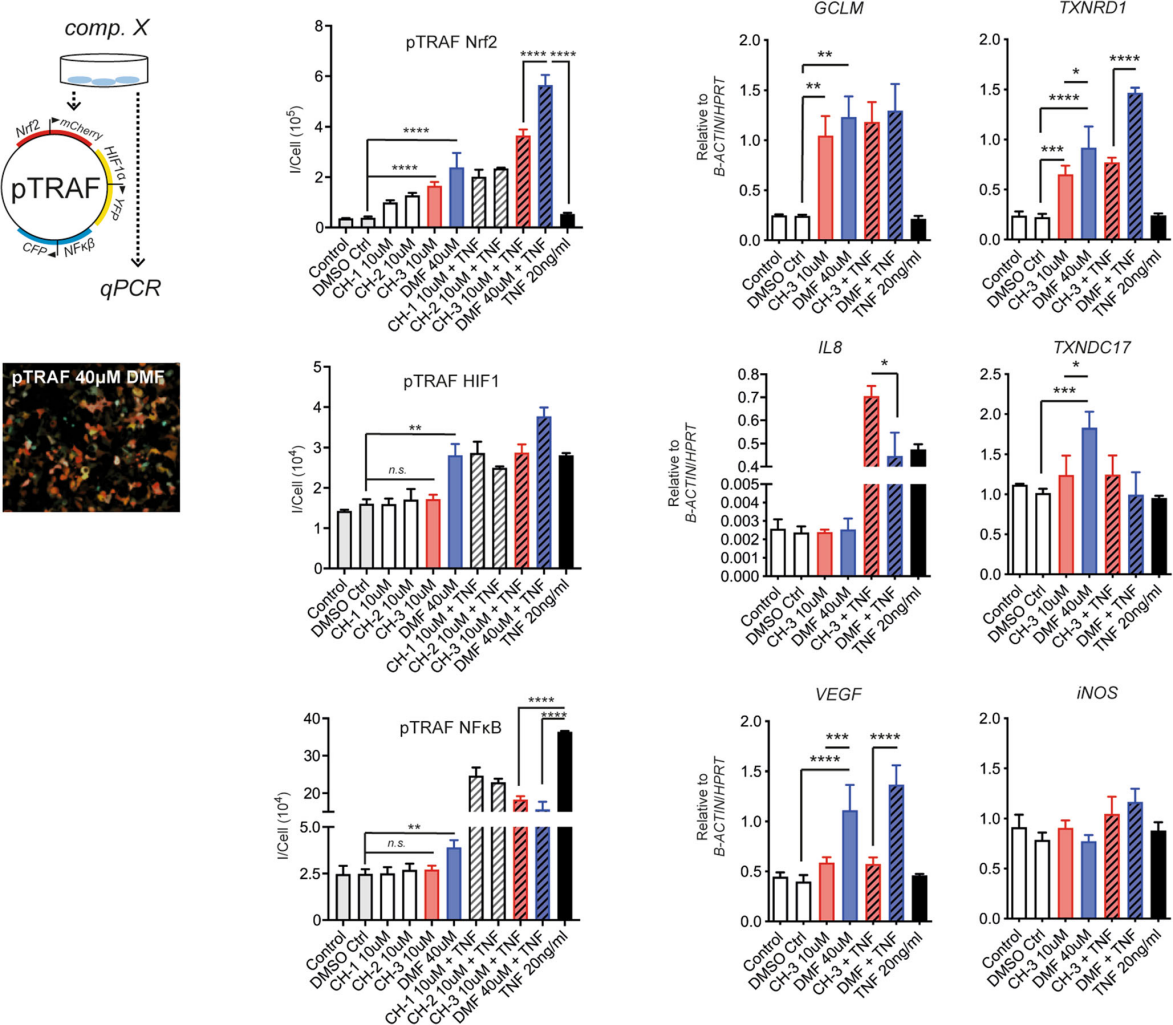
Stable transfected HEK(pTRAF^Nrf2/HIF/NFkB^) reporter cell line enables simultaneous detection of Nrf2, NFκB, and HIF1 activation. (A) Schematic illustration of analysis approach using pTRAF and qPCR. (B–D) Stimulation for 24 h with 10 μM of CH-1, CH-2, and CH-3 or 40 μM DMF, with and without 20 ng/mL TNF (*n* = 3). (E) Examples of transcription levels following 3 h of stimulations with DMF (40 μM), CH-3 (10 μM) with and without TNF (20 ng/mL). Error-bars show S.D. Two group comparisons with a control group were done with one-way ANOVA. *P < 0.05, **P < 0.01, ***P < 0.001, ****P < 0.0001

DMF, which is commonly used in RRMS, mainly activates Nrf2, but is also suggested to have additional off-target effects. Thus, this is an incentive to carry out comparison to our newly synthesized compounds. For this, compounds  (CH-1–CH-8) were initially evaluated in HEK293, stably transfected with plasmids for transcription factor reporter activation based upon fluorescence (pTRAF) [14] (Fig. 1(A)). The pTRAF approach enables simultaneous monitoring of DMF-affected transcription factors Nrf2, NFκB, and HIF1 at a single-cell level. CH-1, CH-2, and CH-3 were selected for further analysis based on their activity towards Nrf2 in combination with their cell viability profile (Fig. S1a, b). Optimal working concentrations were also determined for DMF, based on Nrf2-activation and cell viability (Fig. S1c). CH-1, CH-2, and CH-3 all activated Nrf2 with limited off-target effects on NFκB and HIF1 (Fig. 2(A–D)). In contrast, while DMF activated Nrf2, it also activated HIF1 and downstream transcription of *VEGF* (Fig. 2(B–E)). DMF also caused NFκB activation, but without elevation of downstream *IL8* transcription (Fig. 2(D, E)). Both CH-3 and DMF showed increased Nrf2 activation upon co-stimulation with tumor necrosis factor (TNF) compared to only TNF (Fig. 2(B)). CH-3 was thus selected for further characterization and comparison to DMF (Fig. 1(A–C)).

To investigate downstream effects following transcriptional factor activation, we quantified ten transcripts known to be regulated by Nrf2, NFκB, and/or HIF1. HEK(pTRAF^Nrf2/HIF/NFkB^) cells were stimulated with DMF (40 μM) or CH-3 (10 μM) and among the evaluated transcripts, *TXNRD1, TXNDC17* and *VEGF* appeared to differ between CH-3 and DMF stimulation (Fig. 2(E), Fig. S2). In contrast, transcripts generally associated with Nrf2, such as *GSTA4*, *GCLM*, *HMOX1*, and *NQO1*, did not differ in expression between the two compounds (Fig. S2). This validated the results obtained from the pTRAF reporter system, i.e., that CH-3 displayed a similar capacity as DMF to activate Nrf2 *in vitro*.

### Transcriptional Changes by CH-3 and DMF in Human Cells Are Partially Conserved in Rat Glial Cultures

**Fig. 3:**
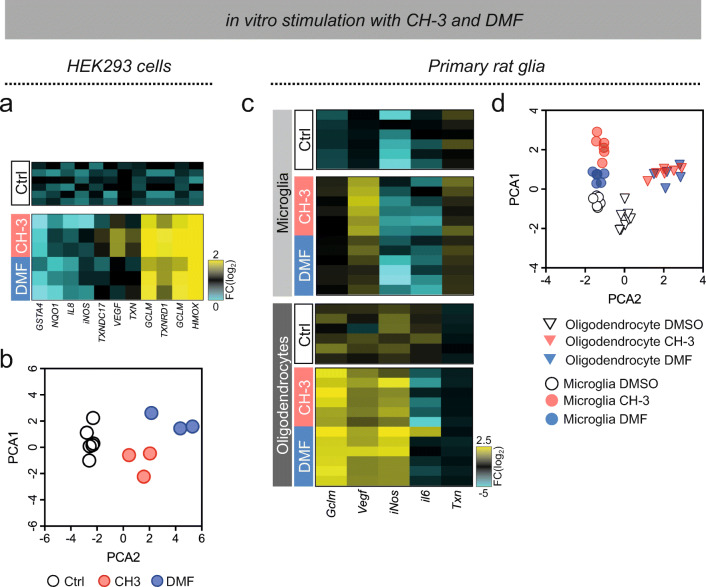
Transcriptional profile in HEK293 cells and primary cultures of microglia and oligodendrocyte following DMF and CH-3. (A) Heat-map of transcript fold-change (Log_2_) following CH-3 and DMF stimulation. (B) PCA reduction plot based on the transcription of genes indicated in the heat-map after 3 h of stimulations with CH-3 (red), DMF (blue), or unstimulated (white). (C) Heat-map of transcription levels following 3 h of stimulations with DMF (15 μM), CH-3 (10 μM) (*n* = 6). (D) PCA reduction plot based on the transcription of genes indicated in the heat-map after 3 h of stimulations (*n* = 6)

To illustrate the transcriptional profiles following CH-3 or DMF stimulation, the expression of transcripts in Fig. 3(A) and Fig. S2 were reduced in a principal component analysis (PCA) plot. This indicated separate clustering of DMF compared to CH-3, and also different from unstimulated samples (Fig. 3(B)). Further, Nrf2 is a suggested target in both microglia and oligodendrocytes (OLs) upon stimulation with vinyl sulfone or DMF, respectively [12, 20, 31]. In order to explore transcriptional patterns downstream of Nrf2, NFκB, and HIF1 and to verify HEK293 results in cell types relevant for the CNS, we established primary rat microglia and OL cultures and exposed them to CH-3 or DMF (Fig. S3a-d). The cultures were stimulated with either DMF (15 μM) or CH-3 (10 μM) for 1 h or 3 h, followed by quantification of transcripts regulated by Nrf2, NFκB, and/or HIF1. In line with the experiments conducted in HEK293 cells, Nrf2-activated transcripts, such as *Gclm*, showed similar regulation with both DMF and CH-3 (Fig. 3(C)). In contrast, *Vegf* and *iNos* clearly differed between DMF, CH-3, and controls (Fig. 3(C)). When including an earlier time point, 1 h following DMF or CH-3 stimulation, this also revealed differences between the two compounds regarding expression in OLs (Fig. S3e). Thus, *Gclm*, *Txn*, and *Il6* all were significantly different between DMF and CH-3 after 1 h of stimulation.

When transcriptional patterns at 3 h were complied into a PCA plot, relatively large differences were recorded between the two cell types (Fig. 3(D)). However, microglia displayed a diverse Nrf2-pattern in response to CH-3 or DMF and thus grouped into two separate clusters away from control stimulation (Fig. 3(D)). In contrast, OLs displayed a more overlapping response to CH-3 and DMF.

### CH-3 and DMF Affects Numbers of Pre-OLs and Neurons after TBI

**Fig. 4:**
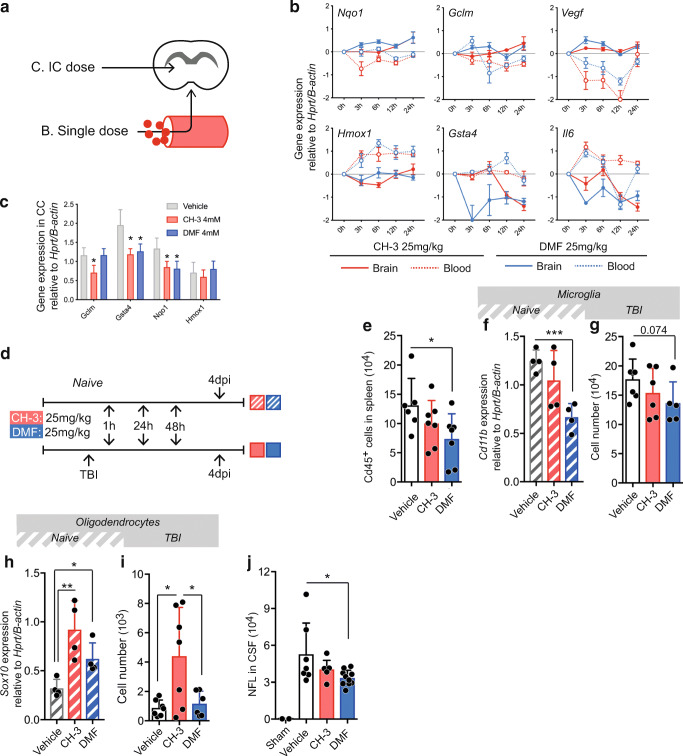
Naïve *in vivo* characterization of DMF and CH-3 and following TBI. (A) Experimental outline for routes of administration. (B) Transcriptional pattern in peripheral blood cells (*dash*) and brain (*solid*). (C) Transcription pattern in corpus callosum (CC) 5 h following intra cisterna injections of DMF (4 mM) and CH-3 (4 mM) (*n* = 5). (D) Experimental outline for TBI in combination with intervention. (E) Flow cytometric valuation of Cd45^+^ (leukocytes) in the spleen compared to vehicle (*n* = 6). (F, G) Microglia in brain following CH-3 or DMF treatment assessed by transcription of *Cd11b* (*n* = 4) (F), and following CH-3 or DMF treatment and TBI (g) assessed by flow cytometry (*n* = 7 + 5). (H, I) Oligodendrocytes (OL) in brain following CH-3 or DMF treatment assessed by the OL lineage marker *Sox10* (*n* = 4) (H), and following CH-3 or DMF treatment and TBI assessed by flow cytometry (*n* = 7) (I). (J) Levels of NFL in the CSF in sham and following TBI in combination with treatment, detected with ELISA. Error-bars show S.D. Two group comparisons with a control group were done with one-way ANOVA. *P < 0.05, **P < 0.01, ***P < 0.001

In RRMS, DMF is administered as an oral tablet preparation. As a first step, we evaluated the capacity of CH-3 and DMF to change brain transcription patterns upon different routes of administration in Dark Agouti (DA) rats (Fig. 4(A)).

To evaluate effects after oral administration, DA rats were given either CH-3 or DMF per body weight or the corresponding volume of vehicle by oral gavage. The dose was decided following titration with increasing doses (Fig. S4a-d). Animals were administered the same dose independent of compound, since similar doses of CH-3 and DMF were effective in primary rat cells (Fig. 3(C, D)). Brain cortex tissue and peripheral blood leukocytes were collected at 3, 6, 12, and 24 h following a single gavage dose. In general, both DMF (25 mg/kg) and CH-3 (25 mg/kg) generated larger transcriptional fluctuations in peripheral blood leukocytes compared to brain tissue (Fig. S5). *Gclm* and *Ark1b8*, both regulated by Nrf2, were elevated in peripheral blood leukocytes following DMF administration, whereas CH-3 had no discernible effect (Fig. S5). However, *Nqo1*, also considered regulated by Nrf2, was elevated in brain following CH-3, but not after DMF administration (Fig. 3(B), Fig. S5). In the brain, CH-3 induced elevated transcription of the Nrf2-regulated genes *Nqo1*, *Hmox1*, and *Gclm*. Interestingly, the temporal expression following DMF was largely similar for these transcripts (Fig. 4(B)).

To confirm transcriptional effects of either compounds in the brain parenchyma, CH-3 (4 mM), DMF (4 mM), or the equivalent volume of vehicle was injected in cisterna magna followed by dissection of corpus callosum (CC) 5 h following the injection. CH-3 and DMF both changed the expression of Nrf2-regulated genes compared to vehicle without discernible differences between the two compounds (Fig. 4(A, C), Fig. S4e).

Nrf2, NFκB, and HIF1 are all activated in responses following TBI and since DMF has been reported to exert beneficial cognitive effects following TBI [4], we repeated that experimental setup to further evaluate CH-3 in direct comparison to DMF (Fig. 4(D)). In brief, rats were administered DMF, CH-3, or corresponding volume of vehicle 1, 24, and 48 h following TBI. The number of all Cd45^+^ cells in spleen was significantly lowered following TBI in combination with DMF compared to vehicle (Fig. 4(E)). However, there were no statistical difference in cell numbers between DMF and CH-3.

The reduction of leukocytes in the spleen could potentially be explained by increased migration to the brain after injury, but we could not observe differences across treatments in brain-infiltrating monocytes or total number of Cd45^+^ cells in the brain (data not shown). In the brain parenchyma, DMF also lowered transcription of the microglia marker *Cd11b* in TBI naïve conditions (administration of drug but no injury) (Fig. 4(F)). Following TBI, DMF suggested a trend for lowered microglia numbers, however not significant (Fig. 4(G)). CH-3 displayed a large variance in TBI naïve conditions and showed no significant effects (Fig. 4(F, G)). When assessing differentiated but pre-myelinating OLs, both CH-3 and DMF increased the transcription of the OL-lineage marker *Sox10* (Fig. 4(H)). In addition, CH-3 also preserved or increased cell numbers after TBI (Fig. 4(I)). Neurofilament light (NFL) is a component of the functional axon of the nerve cell and is released into cerebrospinal fluid (CSF) upon axonal degeneration, and thus acts as a marker of degree of neuro-axonal degeneration [32]. Animals treated with DMF showed modest, but significantly lower NFL concentrations compared to vehicle, while CH-3 did not differ from control (Fig. 4(J)). NFL levels in sham animals were negligible. In summary, potentially therapeutic effects of DMF or CH-3 appeared to be both tissue and cell-type dependent.

## Discussion

We here addressed the *in vitro* effects of seven newly synthesized vinyl sulfoximine compounds, and subsequently evaluated the effects on glial cells of the brain in comparison to DMF, currently in clinical use for RRMS. The purpose of this study was to evaluate the transcriptional activity of DMF in different *in vitro* and *in vivo* models and to compare this with our de novo synthesized compounds focusing on their effects on brain-resident cells. This is crucial since DMF also engages additional transcription factors next to Nrf2 [13, 15].

It is still not unanimously known to what degree beneficial therapeutic effects of DMF can be ascribed its Nrf2-activating effect. Off-target effects of DMF may also contribute. We have previously described the effects of DMF on monocytes and T cells in RRMS, which revealed that DMF exerts prominent oxidizing effects in the systemic compartment, in turn associated with its clinical efficacy [17]. However, DMF has also been shown to have protective effects in experimental disease models, including TBI [4].

Among the newly synthesized vinyl sulfoximine  compounds characterized here, CH-3 showed the most promising effect on Nrf2 when evaluated in *p*TRAF-transfected HEK293 cells. The lowering of NFκB upon addition of DMF to TNF as compared to just TNF underlines the immunosuppressive features described by DMF in mice and man [33, 34]. The cross comparison of transcripts regulated by DMF and CH-3 revealed only partly overlapping responses. Hence, while Nrf2-regulated transcripts, including *GCLM*, *NQO1*, and *GSTA4*, showed similar regulation upon exposure to DMF and CH-3, *VEGF* and *TXNRD1*, both regulated by HIF1, were only affected by DMF. These findings suggest that CH-3 exerts more limited off-target effects compared to DMF in HEK293 cells.

Upon oral administration, the temporal transcriptional profiles for the Nrf2-regualted genes *Nqo1*, *Hmox1*, and *Gclm* were conserved for both CH-3 and DMF, respectively, where DMF indicated an earlier transcriptional elevation compared to CH-3. The increased transcription was in line with the increase in *Nqo1* and *Gclm* after DMF and CH-3 in cultured OLs, indicating that OLs may be one of the brain-resident cells responding to DMF/CH-3. In contrast, microglia during homeostasis *in vitro* displayed a low degree of changes following either DMF or CH-3. However, this may differ if studied during inflammatory or *in vivo* conditions. In this study, primary cultures were studied during non-inflammatory conditions to avoid non-compound-mediated NFκB input on Nrf2 activity.

It is also noteworthy that following oral administration, DMF and CH-3 generated temporal *Il6* and *Vegf* transcription that drastically differed in the brain compared to leukocytes in peripheral blood. This was also true for *Txn* following DMF administration. This finding highlights the need for a more detailed characterization of pharmacodynamics with determination of concentrations in different tissues. The kinetics of these changes also suggested the existence of negative feedback resulting in lowered expression at later time points. Following intrathecal administration to naïve rats, both DMF and CH-3 gave rise to similar expression changes of Nrf2-regulated transcripts compared to vehicle in CC tissue after 5 h. The outcome at this time point is likely to represent an initial drop in Nrf2-regulated gene transcription, as observed for *Hmox1* and *Txn* at 3 h following oral administration. Thus, the temporal dynamics in changes in expression patterns likely are affected by differences in *in vitro* and *in vivo* conditions.

Nrf2, NFκB, and HIF1, as assessed herein with pTRAF, are all key factors in pathological conditions including  TBI [35–37]. Overall, TBI represents a complex inflammatory cascade comprising for example infiltration of immune cells and activation and proliferation of microglia [38], as well as loss of OLs and axonal degeneration [39]. In line with previous publications, DMF lowered the levels of Cd45^+^ cells, however with a considerable variance [17, 33, 34].

Furthermore, administration of DMF has also been associated with limited glutathione depletion, suggesting a direct or indirect neuroprotective action in relation to oxidative stress [4]. Our finding that DMF reduces, albeit modestly, NFL after TBI is in line with this notion. We also found that DMF led to a prominent increase in the expression of *Gclm* and *Gsta4* after oral administration*,* both of which are responsible for intracellular regulation of glutathione. However, the fact that animals treated with CH-3 did not display any clear protective effect on NFL levels in spite of activation of Nrf2 argues that the action of DMF includes additional effects not only depending on activation of Nrf2. On the other hand, we found that CH-3-treated animals displayed a higher number of differentiated but pre-myelinating OLs, which indicates that CH-3 preserves or stimulates the proliferation of pre-OLs following injury. This suggests that CH-3 contributes to the increase in pre-OLs following injury but CH-3 have limited effect to preserve already myelinating OLs immediately after injury. NFκB is present in pre-OLs but absent in mature OLs [40]. In our *in vitro* assessment, CH-3 did not affect NFκB, *Il6*, or *IL8* compared to DMF and this might be a critical aspect why CH-3 animals display a larger population of pre-OLs following TBI compared to DMF-treated animals. Based on this, our data suggests that a limited involvement of NFκB is beneficial for the maintenance or generation of pre-OLs following TBI.

The experiments conducted in primary microglia and OLs cultures extended our observations from the TBI model and clearly highlighted different responses in the two cell types upon exposure to DMF or CH-3. Hence, the response in OLs to CH-3 or DMF was largely similar, while much larger differences were noted in microglia. A possible explanation for this difference is that the transcriptional machinery regulated by the studied transcription factors differ between OLs and microglia. In fact, NFκB has been shown to be low or absent in OLs [40].

## Conclusions

By stepwise assessment of novel vinyl sulfoximine compoiunds and DMF, we demonstrate that one of these, CH-3, display a more Nrf2-specific activating profile compared to DMF. The Nrf2-specific effect is suggested to influence the OL phenotype in terms of promoting proliferation of pre-myelinating cells. In contrast, DMF but not CH-3 reduced concentrations of a nerve cell death biomarker in CSF, suggesting a neuroprotective effect also involve Nrf2-off-target effects. Further studies are needed to extend these observations in order to explore the therapeutic potential of more selective Nrf2 acting compounds in conditions such as demyelination or brain trauma.

## Electronic Supplementary Material

Fig. S1 Nrf2 activity and cell viability (a) Nrf2 activation in pTRAF transfected HEK cells after increasing concentration of CH-1 – CH-8). (b) Viability of cells from the same experiment. (c) Nrf2 activation and cell number. Samples (a, b) were analyzed in triplicates. Graph (c) shows a representative stimulation performed in duplicates. Error- bars show S.D. (PDF 274 kb).

Fig. S2 Transcriptional expression following CH-3 and DMF. Expression, SD and *p-*value for all markers from experiment Fig. 2e (n=3). All analyzes were performed with one-way ANOVA with correction for multiple comparisons. (PDF 21 kb).

Fig. S3 Transcription in primary cell cultures. (a) Transcription of *NFL, Mbp, Gfap* and *Cd11b* in Cd11b+ or A2B5+ sorted primary cultures (n=4). (b) Representative images. (c-d). Transcription of *Hmox1* (as marker for Nrf2 activity) and *Bax* (as marker for decreased viability) in mixed glia cultures consisting of oligodendrocytes and microglia stimulated with increasing concentration of DMF for 3h (n=5) (c) or with increasing concentration of CH-3 for 3h (n=5) (d). (e) Expression and SD of indicated targets following 1 and 3h of CH3 (10μM) or DMF (15μM) stimulation also depicted in Fig. 3c. Error- bars in a, c, d show S.D. Analyzes in e were performed with one-way ANOVA corrected for multiple comparisons. *Red* indicates differences between Ctrl and either stimulation for 1 or 3h. *Blue* indicates differences between time-points for either stimulation. (PDF 337 kb).

Fig. S4 Transcription in rat spleen and brain. (a) Transcription in spleen (n=3), 4h following oral administration of DMF or vehicle. (b) Transcription in brain (n=3), 4h following oral administration of DMF or vehicle. (c) Transcription in spleen (n=3), 4h following oral administration of CH-3 or vehicle. (d) Transcription in brain (n=3), 4h following oral administration of CH-3 or vehicle. (e) Transcription in corpus callosum (CC) following intra cisterna sham injected (n=3), or administration of vehicle (n=5) or CH-3 and DMF (pooled). Error- bars show S.D. (PDF 161 kb).

Fig. S5 Transcription following oral gavage. Transcriptional expression, SD and *p-*value for all markers from experiment Fig. 4b. Two group comparisons with a control group were done with one-way ANOVA. *P<0.05, **P<0.01, ***P<0.001. (PDF 29 kb).

Supplementary Table 1 (PDF 26 kb).

ESM 1 (PDF 1506 kb).

## Abbreviations

BBB: Blood–brain barrier
CSF: Cerebrospinal fluid
DMF: Dimethyl fumarate
HIF1: Hypoxia-inducible factor 1
Keap1: Kelch-like ECH-associated protein 1
MS: Multiple sclerosis
NFkB: Nuclear factor κ-light-chain-enhancer of activated B cells
NFL: Neurofilament light
Nrf2: Nuclear factor (erythroid-derived 2)-like 2
PD: Parkinson’s disease
pTRAF: Plasmid for transcription factor reporter activation based upon fluorescence
RRMS: Relapsing remitting multiple sclerosis
TBI: Traumatic brain injury
TNF: Tumor necrosis factor
