# Supplementary figures and images for: Characterization of More Selective Central Nervous System Nrf2-Activating Novel Vinyl Sulfoximine Compounds Compared to Dimethyl Fumarate
Source: Neurotherapeutics. 2020 May 11;17(3):1142–52. doi: 10.1007/s13311-020-00855-0 (PMC7609514; doi:10.1007/s13311-020-00855-0)

Fig.S1

A Nrf2 activation

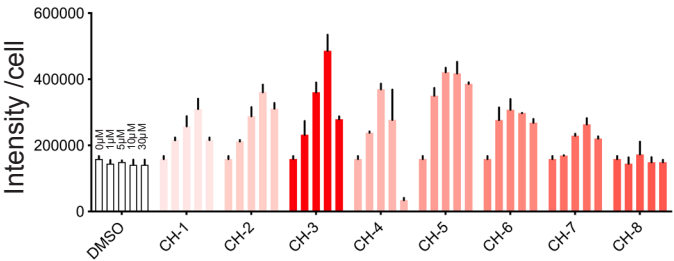

B Viability

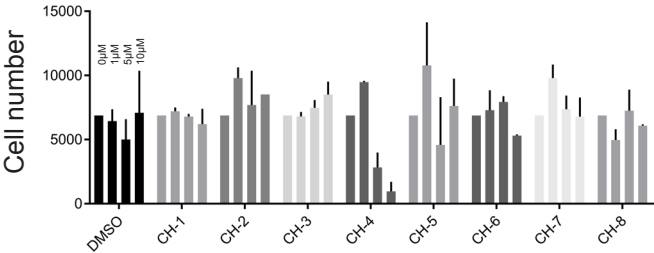

C

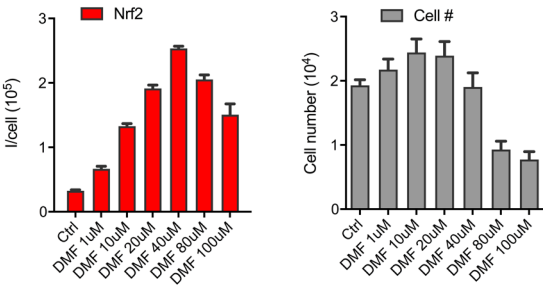

Supplement: Supplementary file 1 — Nrf2 activity and cell viability (a) Nrf2 activation in pTRAF transfected HEK cells after increasing concentration of CH-1 – CH-8). (b) Viability of cells from the same experiment. (c) Nrf2 activation and cell number. Samples (a, b) were analyzed in triplicates. Graph (c) shows a representative stimulation performed in duplicates. Error- bars show S.D. (PDF 274 kb). [file 13311_2020_855_MOESM1_ESM.pdf]

FIG.S4

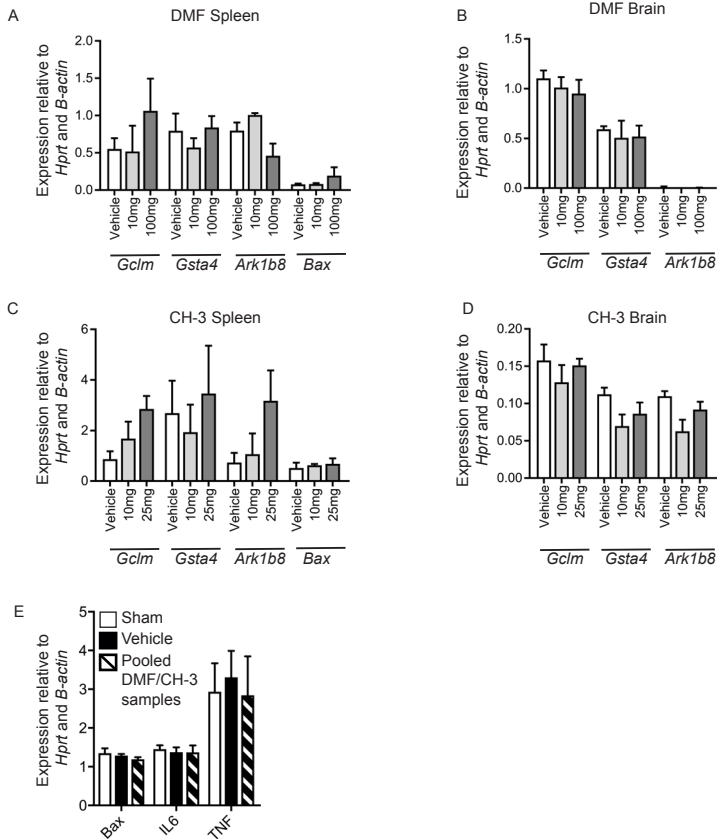

Supplement: Supplementary file 4 — Transcription in rat spleen and brain. (a) Transcription in spleen (n=3), 4h following oral administration of DMF or vehicle. (b) Transcription in brain (n=3), 4h following oral administration of DMF or vehicle. (c) Transcription in spleen (n=3), 4h following oral administration of CH-3 or vehicle. (d) Transcription in brain (n=3), 4h following oral administration of CH-3 or vehicle. (e) Transcription in corpus callosum (CC) following intra cisterna sham injected (n=3), or administration of vehicle (n=5) or CH-3 and DMF (pooled). Error- bars show S.D. (PDF 161 kb). [file 13311_2020_855_MOESM4_ESM.pdf]
