# Supplementary material for: Characterization of More Selective Central Nervous System Nrf2-Activating Novel Vinyl Sulfoximine Compounds Compared to Dimethyl Fumarate
Source: Neurotherapeutics. 2020 May 11;17(3):1142–52. doi: 10.1007/s13311-020-00855-0 (PMC7609514; doi:10.1007/s13311-020-00855-0)
Supplement: Supplementary file 2 — Transcriptional expression following CH-3 and DMF. Expression, SD and p-value for all markers from experiment Fig. 2e (n=3). All analyzes were performed with one-way ANOVA with correction for multiple comparisons. (PDF 21 kb). [file 13311_2020_855_MOESM2_ESM.pdf]

Supplementary Figure 2

	Replicates	Ctrl		DMSO Ctrl		CH-3 (10uM)			DMF (40uM)				CH-3+TNF				DMF+DTNF			TNF (20ng/mL)	
		Expression	SD	Expression	SD	Expression		SD	Expression			SD	Expression			SD	Expression		SD	Expression	SD
GCLM	3	0,24904	0,010	0,24464333	0,011	1,04679	**	0,196	1,23287667	***		0,206	1,18348667	**		0,199	1,29751333	**	0,264	0,21392	0,031
GSTA4	3	1,11876333	0,142	1,06261333	0,076	0,90314		0,200	0,81762333			0,116	1,17741333	***		0,006	1,04509667	**	0,055	0,80019333	0,095
HMOX1	3	0,00628667	0,001	0,00638333	0,001	0,25153	****	0,023	0,24741667			0,019	0,16234333			0,045	0,31505	*	0,192	0,00377667	0,000
IL8	3	0,00258	0,001	0,00238	0,000	0,00239333		0,000	0,00254333			0,001	0,70521667	*	**	0,044	0,44645667	**	0,101	0,4746	0,023
iNOS	3	0,91362667	0,126	0,81026333	0,376	0,78610333		0,218	0,66068667			0,202	1,04754333			0,171	1,39748		0,408	0,88079667	0,083
NQO1	3	0,93200667	0,154	0,78165667	0,050	0,77838		0,093	1,00245333			0,134	1,17880667			0,066	1,18424333		0,133	1,02427	0,007
TRP14	3	1,12076667	0,012	1,01547333	0,054	1,24252333		0,242	1,57546667			0,465	1,24692			0,240	0,99728333		0,278	0,95404667	0,029
TXN	3	0,89254667	0,089	0,93102	0,056	1,17703333		0,205	1,46901667			0,396	1,1948			0,201	1,08030667		0,236	0,86256333	0,002
TXNRD1	3	0,23939	0,041	0,22378	0,034	0,65296333	*	0,086	0,91893333	**		0,212	0,77297333			0,046	1,93603667	*	0,810	0,24131333	0,020
VEGF	3	0,44681333	0,044	0,39995667	0,064	0,58997333	*	0,053	1,11203333	**	*	0,253	0,57502			0,065	1,89441333	*	0,919	0,46095	0,016

one-way ANOVA multipe comparisons

DMSO Ctrl vs. CH-3/DMF vs. DMF	TNF vs. CH-3+TNF/ DMF+TNF	CH-3 vs. DMF or CH-3+TNF vs. DMF+TNF

p-value				
<0.05				
>0.05	*			
>0.01	**			
>0.001	***			
>0.0001	****			
