# Supplementary material for: Characterization of More Selective Central Nervous System Nrf2-Activating Novel Vinyl Sulfoximine Compounds Compared to Dimethyl Fumarate
Source: Neurotherapeutics. 2020 May 11;17(3):1142–52. doi: 10.1007/s13311-020-00855-0 (PMC7609514; doi:10.1007/s13311-020-00855-0)
Supplement: Supplementary file 3 — Transcription in primary cell cultures. (a) Transcription of NFL, Mbp, Gfap and Cd11b in Cd11b+ or A2B5+ sorted primary cultures (n=4). (b) Representative images. (c-d). Transcription of Hmox1 (as marker for Nrf2 activity) and Bax (as marker for decreased viability) in mixed glia cultures consisting of oligodendrocytes and microglia stimulated with increasing concentration of DMF for 3h (n=5) (c) or with increasing concentration of CH-3 for 3h (n=5) (d). (e) Expression and SD of indicated targets following 1 and 3h of CH3 (10μM) or DMF (15μM) stimulation also depicted in Fig. 3c. Error- bars in a, c, d show S.D. Analyzes in e were performed with one-way ANOVA corrected for multiple comparisons. Red indicates differences between Ctrl and either stimulation for 1 or 3h. Blue indicates differences between time-points for either stimulation. (PDF 337 kb). [file 13311_2020_855_MOESM3_ESM.pdf]

FIG.S3

A

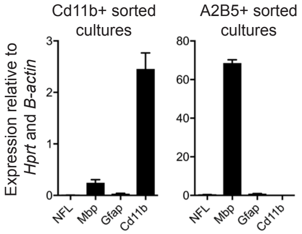

B

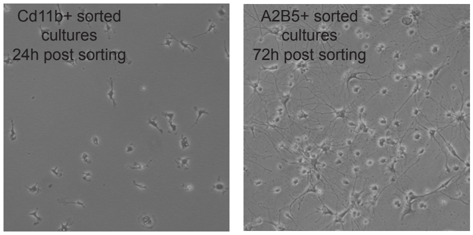

C

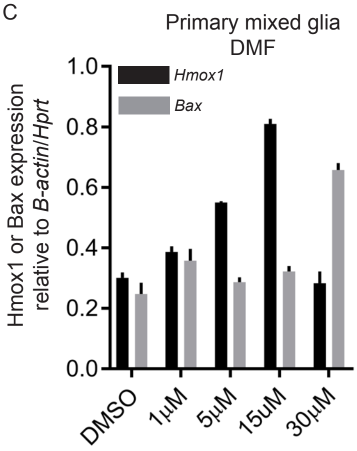

D

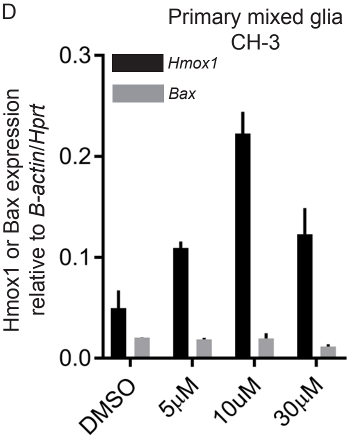

E

Oligodendrocytes

		Ctrl		DMF 1h				DMF 3h				CH-3 1h				CH-3 3h			
	Replicates	Expression	SD	Expression			SD	Expression			SD	Expression			SD	Expression			SD
Gclm	6	2,07739667	0,507	2,11664333	****		0,134	6,570718	****	****	2,353	1,34706667	****		0,507	5,252025	**	****	1,365
Il6	6	1,72675218	0,836	3,36142667	***	****	0,843	0,829435	****		0,268	2,21154333			0,229	0,39536	**	****	0,252
iNos	6	3,94535651	1,631	2,87679333			0,504	6,95067497			4,620	3,53679333			0,608	7,04857667			2,557
Nqo1	6	4,197088	0,545	3,01441	***		0,185	15,9336089	**	***	10,797	3,242675	****		0,301	19,10266	***	****	1,972
Txn	6	0,80136833	0,124	0,787845			0,062	0,80074833			0,084	0,72218667			0,043	0,815055			0,079
Vegf	6	1,44839333	0,527	2,256755			0,221	2,77488			2,853	1,99058			0,254	2,58787			0,383

Microglia

		Ctrl		DMF 1h				DMF 3h				CH-3 1h				CH-3 3h			
	Replicates	Expression	SD	Expression			SD	Expression			SD	Expression			SD	Expression			SD
Gclm	6	0,550857	0,092	0,61724167			0,247	1,51684333	*		0,103	0,53588	*		0,067	1,520335	*	*	1,274
Il6	6	0,84826833	0,330	0,77916167			0,433	0,434345			0,243	0,73966			0,101	0,33553	*		0,325
iNos	6	0,276188	0,128	0,1075425			0,033	0,34935			0,291	0,55764167			0,146	0,4464125			0,473
Nqo1	6	0,99160833	0,061	0,74104833			0,099	1,15078333			0,059	0,79331667			0,070	1,41161833			1,274
Txn	6	1,69032167	0,368	1,429584			0,342	1,58441902			0,671	1,29470167			0,245	2,02706	*		0,456
Vegf	6	0,83591	0,059	1,45325	*		0,060	1,6019564			0,724	1,435735	****		0,229	3,56042833	****	****	0,397

one-way ANOVA multipe comparisons

p-value		Ctrl vs. 1h or 3h	1h vs. 3h
<0.05			
>0.05	*		
>0.01	**		
>0.001	***		
>0.0001	****		
