# Supplementary material for: Characterization of More Selective Central Nervous System Nrf2-Activating Novel Vinyl Sulfoximine Compounds Compared to Dimethyl Fumarate
Source: Neurotherapeutics. 2020 May 11;17(3):1142–52. doi: 10.1007/s13311-020-00855-0 (PMC7609514; doi:10.1007/s13311-020-00855-0)
Supplement: Supplementary file 5 — Transcription following oral gavage. Transcriptional expression, SD and p-value for all markers from experiment Fig. 4b. Two group comparisons with a control group were done with one-way ANOVA. *P<0.05, **P<0.01, ***P<0.001. (PDF 29 kb). [file 13311_2020_855_MOESM5_ESM.pdf]

Supplementary Figure 5

Blood

CH-3 25mg/kg

		ctrl			3h			6h			12h			24h		
	Replicates	Expression	SD	*P*	Expression	SD	*P*	Expression	SD		Expression	SD	*P*	Expression	SD	*P*
Ark1b8	4	0,4158825	0,051		0,502655	0,145		0,5829733	0,013		0,59357	0,168		0,6849867	0,165	
Gclm	4	0,151465	0,027		0,1255075	0,028		0,1237525	0,037		0,10287	0,022		0,1110033	0,013	
Gsta4	4	0,096535	0,019		0,0859475	0,025		0,08187	0,025		0,0689167	0,020		0,07306	0,007	
Nqo1	4	0,10761	0,020		0,0966925	0,039		0,10339	0,013		0,09311	0,009		0,1154	0,007	
Hmox1	4	0,13456	0,009		0,24835	0,053		0,2588575	0,095		0,2583833	0,061		0,2423367	0,022	
iNos	4	2,23376	0,200		0,9413925	0,614	**	1,0784975	0,460	*	1,4952267	0,864		1,11166	0,077	
Vegf	4	0,1034325	0,019		0,0514225	0,029	**	0,050845	0,025	**	0,0624167	0,051	***	0,1176967	0,083	
Txn	4	0,26996	0,015		0,2508125	0,046		0,3109575	0,071		0,28495	0,023		0,192707	0,100	
Il6	4	0,660465	0,275		1,537115	0,441	**	1,028175	0,346		0,9683767	0,331		0,918985	0,078	

DMF 25mg/kg

	ctrl			3h			6h			12h			24h		
	Expression	SD	*P*	Expression	SD	*P*	Expression	SD	*P*	Expression	SD	*P*	Expression	SD	*P*
Ark1b8	0,4158825	0,051		0,65288	0,151	**	0,68785	0,348		0,6232767	0,155		0,6055867	0,233	
Gclm	0,151465	0,027		0,2259333	0,053	**	0,0909067	0,040	**	0,1072567	0,004		0,13127	0,017	
Gsta4	0,096535	0,019		0,1615967	0,028		0,0608133	0,027		0,0799967	0,005		0,08673	0,009	
Nqo1	0,10761	0,020		0,1335325	0,035		0,1421233	0,013		0,1063167	0,007		0,1208467	0,018	
Hmox1	0,13456	0,009		0,2155775	0,086		0,3458167	0,062		0,26893	0,086		0,2736733	0,076	
iNos	2,23376	0,200		1,59128	0,757		2,02096	0,476		1,16866	1,068	**	1,4929467	0,494	
Vegf	0,1034325	0,019		0,0807875	0,024		0,0693667	0,023		0,0451567	0,009	**	0,0820067	0,012	
Txn	0,26996	0,015		0,2871975	0,013		0,3160433	0,074		0,4477767	0,135	**	0,22559	0,052	
Il6	0,660465	0,275		1,2546675	0,276	*	0,9775233	0,276		0,26563	0,041		0,7867167	0,176	

Brain

CH-3 25mg/kg

		ctrl			3h			6h			12h			24h		
	Replicates	Expression	SD	*P*	Expression	SD	*P*	Expression	SD	*P*	Expression	SD	*P*	Expression	SD	*P*
Ark1b8	4	0,05796	0,004		0,05885	0,012		0,0776425	0,028		0,0489867	0,011		0,0740767	0,016	
Gclm	4	0,0372775	0,004		0,0442767	0,001		0,047245	0,012		0,0337	0,005		0,04679	0,010	
Gsta4	4	0,0788075	0,007		0,0784725	0,016		0,0816833	0,009		0,0678967	0,007		0,068335	0,006	
Nqo1	4	0,1167075	0,019		0,118275	0,015		0,118185	0,030		0,13787	0,007		0,1844133	0,050	**
Hmox1	4	0,05018	0,008		0,0384625	0,005		0,0365	0,006		0,05015	0,010		0,0597633	0,016	
iNos	4	0,0221967	0,018		0,020845	0,018		0,0342	0,030		0,00927	0,000		0,0181313	0,006	
Vegf	4	1,7060825	0,180		2,014995	0,122		1,9593725	0,208		1,8229333	0,204		2,1861233	0,122	**
Txn	4	1,626855	0,108948		1,61586	0,468872		2,056155	0,71144		0,8676067	0,160846	*	0,6305467	0,134692	**
Il6	4	1,630505	0,421234		1,286835	0,475443		1,9331225	0,691454		0,8535667	0,138115		0,6133533	0,131913	

DMF 25mg/kg

	ctrl			3h			6h			12h			24h		
	Expression	SD	*P*	Expression	SD	*P*	Expression	SD	*P*	Expression	SD	*P*	Expression	SD	*P*
Ark1b8	0,05796	0,004		0,04905	0,014		0,048895	0,019		0,06466	0,006		0,051405	0,028	
Gclm	0,0383767	0,004		0,0347	0,006		0,0374967	0,006		0,04155	0,001		0,0530333	0,020	
Gsta4	0,0788075	0,007		0,08208	0,010		0,06504	0,009		0,07844	0,005		0,0874267	0,023	
Nqo1	0,1167075	0,019		0,146205	0,011		0,15917	0,015		0,1394033	0,014		0,1854467	0,060	
Hmox1	0,05018	0,008		0,0429875	0,014		0,05345	0,013		0,0511567	0,010		0,0455333	0,006	
iNos	0,0221967	0,018		0,02131	0,007		0,01723	0,001		0,01003	0,003		0,0123867	0,006	
Vegf	1,7060825	0,180		0,29612	0,017	*	2,3067333	0,255	***	1,6620533	0,049		2,11362	0,210	*
Txn	1,626855	0,108948		0,04704	0,014142	**	1,6747	1,517477		0,82526	0,263303	*	0,7196067	0,111189	*
Il6	1,630505	0,421234		0,63321	0,070711		1,81035	1,351243		0,9618567	0,535722		0,8635367	0,223242	

SD = Standard deviation

P = Two group comparisons with a control group were done with one-way ANOVA. *P<0.05, **P<0.01, ***P<0.001.
