# Supplementary material for: Characterization of More Selective Central Nervous System Nrf2-Activating Novel Vinyl Sulfoximine Compounds Compared to Dimethyl Fumarate
Source: Neurotherapeutics. 2020 May 11;17(3):1142–52. doi: 10.1007/s13311-020-00855-0 (PMC7609514; doi:10.1007/s13311-020-00855-0)
Supplement: Supplementary file 6 — (PDF 26 kb). [file 13311_2020_855_MOESM6_ESM.pdf]

Supplementary Table 1

	DMF	CH-1/VSC-2	CH-2	CH-3	CH-4	CH-5	CH-6	CH-7	CH-8
molMW	144.13	308.78	307.79	321.82	332.80	403.80	413.88	383.89	418.34
QPlogPo/w	0.21	3.04	2.57	3.16	2.32	3.32	2.28	4.78	5.15
PSA	75.4	42.9	47.4	34.7	60.2	60.1	81.2	36.5	22.9
QPlogS	-0.935	-3.343	-1.865	-2.033	-1.739	-2.557	-1.677	-4.069	-4.319
QPPCaco	553	1876	62	168	38	89	40	169	178
QPlogBB	-0.786	-0.248	-0.391	0.037	-0.709	-0.044	-0.826	-0.026	0.114
HumanOralAbsorption	3	3	3	3	3	3	3	3	3
CNS	-1	0	0	1	-1	-1	-1	0	1
#stars	4	0	0	0	1	0	1	1	0

Selected properties calculated using: Schrödinger Release 2019-4: QikProp, Schrödinger, LLC, New York, NY, 2019

molMW	*molecular weight*
QPlogPo/w	*predicted octanol/water partition coefficient*
PSA	*van der Waals surface of polar N, O, and carbonyls*
QPlogS	*predicted aqueous solubility, log S; S in mol/dm3*
QPPCaco	*predicted apparent Caco2 permeability in nm/sec; >25 ok*
QPlogBB	*predicted brain-blood partition coefficient*
HumanOralAbsorption	*predicted human oral absorption 1-3; 3 = high*
CNS	*predicted CNS activity; -2 (inactive) to 2 (active)*
#stars	*Number of properties that fall outside 95% of known drugs; 0-5 where larger number is less druglike*
